# Improving robustness of 3D multi-shot EPI by structured low-rank reconstruction of segmented CAIPI sampling for fMRI at 7T

**DOI:** 10.1016/j.neuroimage.2022.119827

**Published:** 2023-02-15

**Authors:** Xi Chen, Wenchuan Wu, Mark Chiew

**Affiliations:** a Wellcome Centre for Integrative Neuroimaging, FMRIB, Nuffield Department of Clinical Neurosciences, University of Oxford, Oxford, United Kingdom; b Physical Sciences, Sunnybrook Research Institute, Toronto, Canada; c Department of Medical Biophysics, University of Toronto, Toronto, Canada

**Keywords:** 3D fMRI, 7T fMRI, Multi-shot EPI, Physiological noise, CAIPI sampling, Structured low-rank reconstruction, Block-Hankel structured matrix

## Abstract

Three-dimensional (3D) encoding methods are increasingly being explored as alternatives to two-dimensional (2D) multi-slice acquisitions in fMRI, particularly in cases where high isotropic resolution is needed. 3D multi-shot EPI acquisition, as the workhorse of 3D fMRI imaging, is susceptible to physiological fluctuations which can induce inter-shot phase variations, and thus reducing the achievable tSNR, negating some of the benefit of 3D encoding. This issue can be particularly problematic at ultra-high fields like 7T, which have more severe off-resonance effects. In this work, we aim to improve the temporal stability of 3D multi-shot EPI at 7T by improving its robustness to inter-shot phase variations. We presented a 3D segmented CAIPI sampling trajectory (“seg-CAIPI”) and an improved reconstruction method based on Hankel structured low-rank matrix recovery. Simulation and in-vivo results demonstrate that the combination of the seg-CAIPI sampling scheme and the proposed structured low-rank reconstruction is a promising way to effectively reduce the unwanted temporal variance induced by inter-shot physiological fluctuations, and thus improve the robustness of 3D multi-shot EPI for fMRI.

## Highlights

• We proposed an image reconstruction method based on Hankel structured low-rank matrix completion and a 3D segmented CAIPI sampling pattern, which can improve the robustness to physiological fluctuations of 3D multi-shot EPI acquisitions for fMRI at 7T.
• The proposed method can boost the tSNR of 3D EPI imaging and make it comparable or even higher than the tSNR of 2D SMS-EPI imaging, when the latter is significantly higher in physiological noise dominated regimes.
• The proposed method reconstructs each volume independently and can preserve the full temporal degree of freedom of the time series.

## 1 Introduction

Three-dimensional (3D) encoding methods are increasingly being explored as alternatives to two-dimensional (2D) simultaneous multi-slice EPI (SMS-EPI) acquisitions in functional MRI (fMRI). Compared to conventional 2D multi-slice imaging, 3D imaging is well known to provide SNR benefits (Poser et al., 2010) as the whole volume rather than a thin slice is excited repeatedly for every shot. Also, 3D imaging is less likely to run into SAR issues due to the much lower optimal flip angles compared to SMS-EPI. In addition, 3D imaging can achieve high isotropic resolution without being limited by RF slice profiles along the slice encoding direction. All these advantages contribute to the prevalence of 3D encoding in high isotropic resolution fMRI, which has received strong interests for applications such as layer-specific fMRI (Lawrence et al., 2019).

3D multi-shot EPI, which samples a 2D $k_{x}$-$k_{y}$ plane for each shot is one of the most popular choices for 3D fMRI. However, the main disadvantage of 3D multi-shot EPI imaging, compared to 2D acquisitions which use a single-shot readout like SMS-EPI, is that it suffers from increased vulnerability to shot-to-shot inconsistencies due to physiological fluctuations. The tSNR of a time series, defined as its temporal mean divided by the temporal standard deviation, reflects the temporal stability of a given time course, and relates to the sensitivity to subtle activations of the fMRI measurement. In the absence of any temporal fluctuations like physiological noise or scanner instabilities, tSNR is the same as the image SNR of each volume. In practice, physiological noise, which scales with the signal intensity, creates an asymptotic limit on the achievable tSNR (Krüger and Glover, 2001, Triantafyllou et al., 2005, Triantafyllou, Hoge and Wald, 2006). Previous work has shown that this tSNR limit depends on the number of shots used for a conventional 3D multi-shot EPI acquisition, and that a larger number of shots is usually associated with a lower tSNR at the same SNR level (van der Zwaag et al., 2012), since the shot-to-shot k-space inconsistencies can result in temporally varying ghost artifacts and thus a reduction in tSNR. Hence, the SNR benefits enabled by 3D encoding are not fully realized for 3D multi-shot EPI acquisition, and it might only be able to offer higher tSNR than 2D single shot EPI in low SNR, thermal noise dominated regimes (Jorge et al., 2013; Lutti et al., 2013). Thus, a typical choice is to use 3D acquisition at 1 mm isotropic resolution or smaller voxel sizes, and SMS-EPI for bigger voxel sizes. Previous work (Huber et al., 2018) has showed that the tSNR curves for 3D EPI and 2D SMS-EPI crossed at around 1 mm isotropic resolution for BOLD fMRI at 7T.

The physiological fluctuations which trouble 3D multi-shot EPI acquisition can manifest themselves as rather localized fluctuations of blood and cerebrospinal fluid related to cardiac pulsation, and spatially varying phase modulations resulting from the B0 fluctuations mainly caused by the movement of the chest during respiration (Tijssen et al., 2011; Zahneisen et al., 2014), the latter of which is particularly troublesome for multi-shot imaging. Also, as the off-resonance effects scale with field strength, the physiologically induced inter-shot phase variations can be more detrimental at ultra-high fields like 7T, which plays an important role in high resolution fMRI due to its ability to boost SNR in small voxel regimes.

While various methods have been proposed to reduce physiological noise for fMRI, most of them are post-processing methods, either “model-based” (Agrawal et al., 2020; Glover et al., 2000; Hutton et al., 2011; Jorge et al., 2013; Kasper et al., 2017; Lutti et al., 2013; Tijssen et al.,2014) or “data-driven” (Jorge et al., 2013; Kasper et al., 2017; Salimi-Khorshidi et al., 2014; Behzadi et al., 2007), that work on reconstructed image time series. Unlike these post-processing methods, reconstruction methods which take into account the characteristics of multi-shot acquisitions generally rely on navigators to estimate the shot-to-shot phase variations (Barry et al., 2008; Barry and Menon, 2005). However, navigator techniques estimate dynamic phase information at the cost of prolonged acquisition time and reduced temporal resolution, and it can be particularly challenging to acquire 3D navigators. In the context of diffusion imaging, motion induced inter-shot phase variations due to the use of strong diffusion encoding gradients present a similar problem for multi-shot EPI acquisitions, and a variety of methods have been proposed to deal with this issue. These methods in general are based on explicit phase estimates (Atkinson et al., 2006, Chen et al., 2013, Porter and Heidemann, 2009). Recently, image reconstruction methods based on Hankel structured low-rank matrix completion (Haldar and Setsompop, 2020; Jacob et al., 2020) have been proposed to resolve the odd/even echo phase differences (Lee, Jin and Ye, 2016a, Lobos et al., 2018, Lobos et al., 2021) as well as the shot-to-shot phase variations for EPI acquisitions (Mani et al., 2017). Specifically, the MUSSELS method (Mani et al., 2017) originally proposed for multi-shot diffusion data has demonstrated its ability to account for inter-shot motion induced phase variations without requiring explicit knowledge of them, and has shown superior performance compared to explicit phase estimation methods. While few applications of structured low-rank reconstructions have focused on fMRI, the ALOHA method (Lee et al., 2016a) and the RAC-LORAKS method (Lobos et al., 2021) evaluated Nyquist-ghost corrections in fMRI or fMRI-like experiments.

This work aims to improve the temporal stability of 3D multi-shot EPI for fMRI at 7T, at isotropic spatial resolutions between 1.0 mm and 1.8 mm, by reducing its vulnerability to physiologically induced inter-shot phase variations. We propose a reconstruction method based on Hankel structured low-rank matrix recovery, using an adaptation of the MUSSELS approach, to reduce the impact of physiologically induced k-space inconsistencies in 3D multi-shot EPI. To adapt to the proposed reconstruction, a 3D CAIPI sampling trajectory with interleaved ordering along $k_{z}$ was also used. Both simulation and in-vivo experiments demonstrate that the combination of the interleaved CAIPI sampling trajectory and the structured low-rank reconstruction method provides a robust way to improve the tSNR of 3D multi-shot EPI imaging for fMRI.

## 2 Methods

An ideal and straightforward solution to reduce vulnerability to shot-to-shot inconsistencies is to reconstruct an individual image for each shot and perform a phase-insensitive shot combination in image space. However, as 3D whole brain EPI acquisitions typically consist of a large number of shots, we propose to bin multiple consecutive shots together into shot groups, and perform joint reconstruction for each shot group instead to improve the conditioning of the reconstruction. Also, along with the proposed reconstruction, a 3D blipped-CAIPI readout with interleaved acquisition ordering along $k_{z}$ referred to as “seg-CAIPI” is used to optimize the sampling pattern for each shot group. The seg-CAIPI sampling and the joint reconstruction will be demonstrated in detail in the following sections.

### 2.1 Seg-CAIPI sampling trajectory

**Fig. 1:**
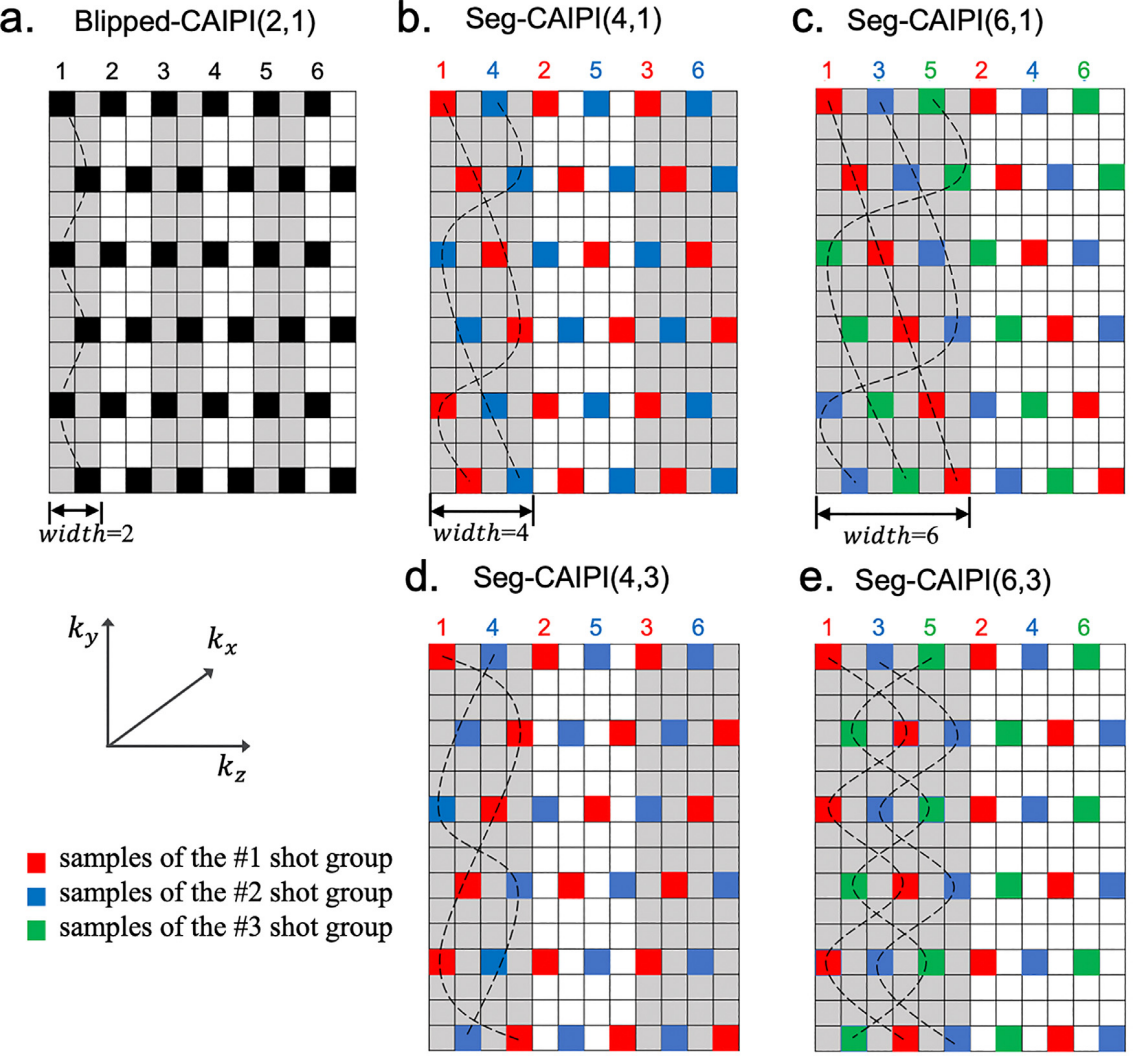
Schematic 3D multi-shot EPI sampling patterns. (a) Standard blipped-CAIPI. (b)–(e) The proposed seg-CAIPI scheme with varying choices of width and $\Delta k_{z}$ blip. The sampling patterns are shown in $k_{y}$-$k_{z}$ plane and each sample represents a readout line. All the samples connected by a dashed line correspond to a single shot. Different shot groups are marked in different colours. The under-sampling factor is R=3×2. For seg-CAIPI sampling patterns with width=4 in (b) and (d), 2 shot groups are used. For seg-CAIPI sampling patterns with width=6 in (c) and (e), 3 shot groups are used.

CAIPI sampling patterns have reduced g-factors compared to conventional non-CAIPI sampling (Breuer et al., 2005; Setsompop et al., 2012), and 2D CAIPIRINHA have been widely used in 3D EPI acquisitions (Breuer et al., 2006). A conventional 3D blipped-CAIPI multi-shot EPI sampling pattern (Narsude et al., 2016) is shown in Fig. 1a, which uses a sequential ordering along the second phase encoding direction, and it is referred to as the “standard” trajectory in this work. Here, we introduce a 3D segmented CAIPI sampling pattern termed “seg-CAIPI”, which uses interleaved sampling along $k_{z}$, combined with a $k_{z}$-blipped CAIPI pattern as is shown in Fig. 1b–e. Note unlike some previous works (Hendriks et al., 2020, Stirnberg and Stocker, 2021), here the segmentation and interleaved ordering are introduced along $k_{z}$ direction instead of along $k_{y}$ direction for each $k_{z}$ plane.

We use the parameter “width” to describe the offset along $k_{z}$ between two consecutive shots with identical trajectories. For the standard trajectory, its width is the same as $R_{z}$ – the under-sampling factor along the shot dimension. For seg-CAIPI, its width is now $R_{z}\times N$, since interleaved ordering along $k_{z}$ with *N* interleaves is used. Thus, k-space is traversed *N*-fold faster along $k_{z}$. The span of each shot along $k_{z}$ for seg-CAIPI is also defined by the width parameter, indicating an *N*-fold wider span in accordance with the *N*-fold larger offset between two consecutive shots. Note this allows for a $\Delta k_{z}$ blip size $\geq R_{z}$. The next interleave traverses the k-space again to acquire missing samples with a complementary trajectory, which has the same $\Delta k_{z}$ blip size and $k_{z}$-span but is offset by $R_{z}$ along $k_{z}$. Each specific standard blipped-CAIPI and seg-CAIPI trajectory are marked by $\left(\text{width},\Delta k_{z}\right)$. Although different $\Delta k_{z}\;$ blip sizes might be chosen for the standard and the seg-CAIPI schemes, the overall sampling masks are designed to be exactly the same. Typically, all of the shots corresponding to an interleave are binned as a shot group. In the example shown in Fig. 1, each shot group consist of three consecutive shots in Fig. 1b and d, and two shots in Fig. 1c and e respectively. In general, each shot group has a more uniform under-sampling pattern for the seg-CAIPI scheme, while for the standard trajectory, each shot group if defined in the same way has a more localized sampling pattern.

### 2.2 Data acquisition

All in-vivo data were collected on a Siemens Magnetom 7T scanner (Siemens Healthineers, Erlangen, Germany) equipped with a 32-channel head-only receive coil with single channel transmit (Nova Medical, Wilmington, MA, USA). Under-sampling was applied along two dimensions with the total acceleration factor $R=R_{y}\times R_{z}$, where $R_{y}$ and $R_{z}$ denote the acceleration along the primary phase encoding dimension and the shot dimension respectively.

### 2.3 Low-rank constrained reconstruction

**Fig. 2:**
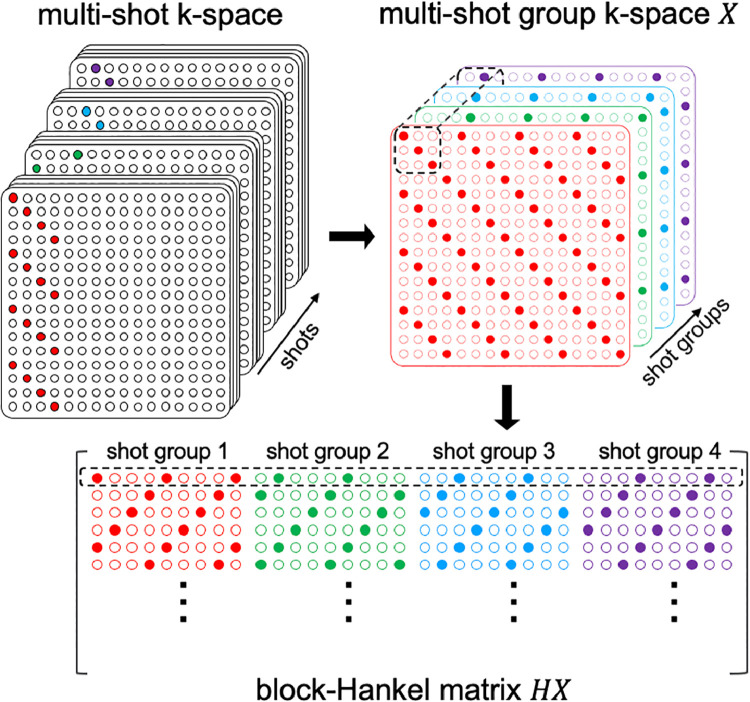
Illustration of the shot-binning and the block-Hankel structured matrix construction from multi-shot group k-space data. A small number of shots acquired consecutively are binned together as a shot group. X denotes the multi-shot group k-space data and HX gives its block-Hankel structured matrix representation. A small patch of X selected by a sliding window is vectorized to generate a row of the block-Hankel matrix.

Conventional reconstruction methods combine all the acquired shots in k-space directly regardless of the shot-to-shot inconsistencies. To alleviate the intra-volume inconsistencies, we aim to reconstruct individual images for each shot group which consists of fewer shots. The images of all the shot groups are then sum-of-squares combined to avoid phase cancellation effects. Similar to the Hankel structured low-rank matrix recovery method MUSSELS, the missing k-space data of all the shot groups are jointly recovered by exploiting the linear dependency among different shot groups, assuming the images for each shot group have the same magnitude and differ only by a physiologically induced phase modulation. To enable this, we ignore intra-shot group variations, as respiratory phase effects (∼3-5s period) are expected to be relatively temporally coherent with respect to the shot TR timescale. This redundancy in multi-shot group k-space can be expressed as the low rank property of its block-Hankel structured matrix representation. The reader is referred to MUSSELS for a more detailed description of this property in multi-shot imaging. The structured low-rank matrix formulation also leverages all other linear inter-dependencies such as those arising from limited image support (Haldar, 2014; Haldar and Zhuo, 2016) simultaneously. Fig. 2 shows one way to construct a block-Hankel structured matrix from the multi-shot group k-space. The image reconstruction is formulated as a low-rank constrained parallel imaging optimisation problem:

(1) $$\underset{X}{\mathrm{argmin}}\frac{1}{2}{\left|\left|\mathrm{EX}-Y\right|\right|}_{2}^{2}+\lambda {\left|\left|\mathrm{HX}\right|\right|}_{*}$$

where X is the k-space of all the shot groups to be reconstructed and Y is the measured multi-coil multi-shot group k-space data. The operator E performs the composition of $M\cdot F\cdot S\cdot F^{-1}$, where F and $F^{-1}$ are the Fourier and inverse Fourier transform respectively. S denotes the multi-coil sensitivity encoding operator, and M selects the sampled k-space locations for each shot group. The operator H applied to X generates the block-Hankel structured matrix of X. ${\left|\left|HX\right|\right|}_{*}\;$ denotes the nuclear norm of HX, which is the convex approximation of rank(HX). λ is the regularisation parameter which weights the low-rank constraint. Eq. (1) is solved using the Alternating Direction Method of Multipliers (ADMM) (Boyd, 2010) algorithm, which reformulates it as a constrained problem of the form:

(2) $$\begin{array}{ccc} & & \underset{X,\;Z}{\mathrm{argmin}}\frac{1}{2}{\left|\left|\mathrm{EX}-Y\right|\right|}_{2}^{2}+\lambda {\left|\left|Z\right|\right|}_{*}\\ & & s.t.\;Z=\mathrm{HX} \end{array}$$

Note the optimisation of a nuclear norm term generally needs a step of soft thresholding on the singular values. In this work, we used hard thresholding instead, which has shown better performance in this problem especially when the tSNR is low.

All reconstructions were implemented in MATLAB R2020a (MathWorks, Inc.). Coil sensitivity maps were calculated using ESPIRiT implemented in the BART toolbox (Uecker et al., 2014). The Nyquist ghosting was corrected using the 3-line reference scan averaged cross all the shots to perform a conventional linear phase correction. The number of shot groups used for the proposed reconstruction was set to be the same as the number of interleaves ($\text{width}/R_{z}$), to achieve roughly uniform sampling for each shot group. The kernel size for the Hankel transform was chosen empirically to be 6×6, and we used a fixed number of 10 iterations for the ADMM optimization. The multi-coil, multi-shot acquired k-space was firstly combined across shots and then combined across coils using the coil sensitivity maps to get the single coil, shot-combined k-space data, which is used as the initialization of the k-space for every shot group. Since image artifacts arising from shot-to-shot inconsistencies do not occur in the readout direction, an inverse Fourier transform was performed along the fully sampled readout direction, followed by separate reconstructions of each 2D $k_{y}$-$k_{z}$ slice, which reduced the computational burden. Reconstruction hyperparameters were chosen empirically to maximize the resulting tSNR of a single sagittal slice and then used for all the slices. λ=3E−3 was used for the simulation datasets and λ=3E−4or6E−4 were used for the in-vivo datasets. Data acquired with the standard trajectory are not compatible with the proposed reconstruction due to the clustered nature of sequentially acquired shots (see supplementary Fig. S1 for example), and were reconstructed by conventional methods, either a direct inverse Fourier Transform on fully sampled data, or SENSE reconstruction (Pruessmann et al., 1999) on under-sampled data.

We evaluated the performance of the proposed method which uses the low-rank constrained reconstruction on the seg-CAIPI sampling data, by comparing to the conventional method which uses the standard blipped-CAIPI sampling with SENSE reconstruction, as well as the 2D SMS-EPI using the CMRR multi-band EPI sequence and online reconstruction (Moeller et al., 2010). We note that the online reconstruction of the CMRR SMS-EPI sequence may yield better tSNR or z-statistic values due to the optimized image reconstruction and data processing pipeline.

### 2.4 Code and data availability statement

All reconstruction code is available at https://github.com/XChen-p/Multishot-EPI.

### 2.5 Simulation experiments

Multi-shot k-space datasets consisting of a single $k_{y}$-$k_{z}$ plane were synthesized by modulating a ground truth image with a series of physiologically plausible phase variation maps, which were measured from a 2D EPI time series acquired in sagittal orientation at 7T with 1.5mm isotropic resolution and TE/TR=20/40ms. The measured phase variation maps were then fit to a second order spherical harmonic basis set for denoising. The k-space data for each shot was generated by resampling the 2D k-space time-series for both the standard trajectory and the seg-CAIPI trajectory. To simulate low/medium/high thermal noise to physiological noise ratios, different levels of complex, zero-mean Gaussian noise were then added to the above k-space data respectively. The physiological noise-free datasets with the same amount of Gaussian noise as the phase variations corrupted datasets were also generated as the reference datasets, with mean SNRs of approximate 97/65/43 respectively, to indicate the tSNR upper bounds of the physiologically corrupted datasets. The performance of the proposed method with seg-CAIPI(6,1) sampling was validated across different thermal to physiological noise ratios in the fully sampled regime. At the medium thermal noise level, the proposed method with different choices of width were also compared where the $\Delta k_{z}$ blip was kept to be 1. In addition, the performance of the proposed method at varying acceleration factors were also investigated at the medium thermal noise level, where seg-CAIPI(6,2) was used for the proposed method at R=1×3 and 3×1, and blipped-CAIPI(3,2) was used for the conventional method at R=1×3. Lastly, the proposed method with seg-CAIPI(8,2) sampling and conventional method with blipped-CAIPI(4,2) sampling are compared in detail at R=2×4 in a thermal noise-free regime*.*

### 2.6 In-vivo experiments

All subjects were scanned with informed consent under a technical development protocol approved by the local ethics committee. Resting-state and task-based fMRI datasets were acquired on seven healthy volunteers to validate the performance of the proposed method. We evaluated the performance of different methods at varying spatial resolutions (1/1.2/1.5/1.8 mm isotropic resolution) and acceleration factors where the ratio between thermal noise and physiological noise changes. We also investigated into the impact of width used for the proposed method which trades off between intra-shot group consistency and the data available for each shot group. Each resting-state dataset consisted of 40 or 60 volumes. A task fMRI experiment was performed on six of the subjects to assess the impact of the proposed approach on BOLD activation. Each subject was scanned twice with both the proposed 3D EPI and SMS-EPI acquisitions in a counterbalanced order, and each scan was approximately 4 minutes with 170 volumes. The experiment used a 30/30s off/on block design, and the subjects were instructed to perform a finger tapping task with both hands when a 10 Hz black and white flashing checkerboard was shown every 30 seconds. The functional datasets were processed using FEAT (Jenkinson et al., 2012; Woolrich et al., 2001), and minimal data pre-processing was used which includes high pass filtering and motion correction by MCFLIRT (Jenkinson et al., 2002).

All in-vivo scanning protocols are shown in Table 1. Slice orientation was transversal with phase encoding along the anterior-posterior direction. A slab-selective sinc excitation was used without slice oversampling. All standard, seg-CAIPI trajectory data and SMS-EPI data used for comparison were acquired with matched TE/TR/bandwidth. Ernst angles for 3D EPI and SMS-EPI were used respectively. For a given width, the $\Delta k_{z}$ blip size was chosen following the principle of minimizing the largest distance between two samples.

**Table 1:** The scanning protocols of the in-vivo experiments.

iso. res. ($mm^{3}$)	matrix size	BW (Hz/pixel)	$R_{y}$	$R_{z}$/ MB factor	TE (ms)	volume TR (ms)	shot TR (ms)	flip angle	FOV shift	echo spacing (ms)	sampling	# shots/shot group
1.8	116×116×96	2052	2/4	2	23	2640	55	15	-	0.57/0.59	blipped-CAIPI(2,1)	48
										0.65	seg-CAIPI(8,3)	12
		2156	2	4	23	1320	55	15	-	0.59	blipped-CAIPI(4,2)	24
										0.63	seg-CAIPI(8,2)	12
							-	64	FOV/4	0.61	SMS-EPI	-
1.5	140×140×96	1880	3	2	24	2592	54	15	-	0.64	blipped-CAIPI(2,1)	48
										0.72	seg-CAIPI(8,3)	12
							-	78	FOV/6	0.68	SMS-EPI	-
		1880	3	3	24	1728	54	15	-	0.66	blipped-CAIPI(3,2)	32
										0.7	seg-CAIPI(6,2)	16
							-	70	FOV/12	0.68	SMS-EPI	-
1.2	174×174×120	1596	3	2	29	3900	65	16	-	0.73	blipped-CAIPI(2,1)	60
										0.75-0.81	seg-CAIPI(4,1), (6,3), (8,3)	30/20/15
							-	85	FOV/6	0.77	SMS-EPI	-
1	210×210×120	1254	4	2	32	4080	68	15	-	1	blipped-CAIPI(2,1)	60
										1	seg-CAIPI(6,3)	20

## 3 Results

**Fig. 3:**
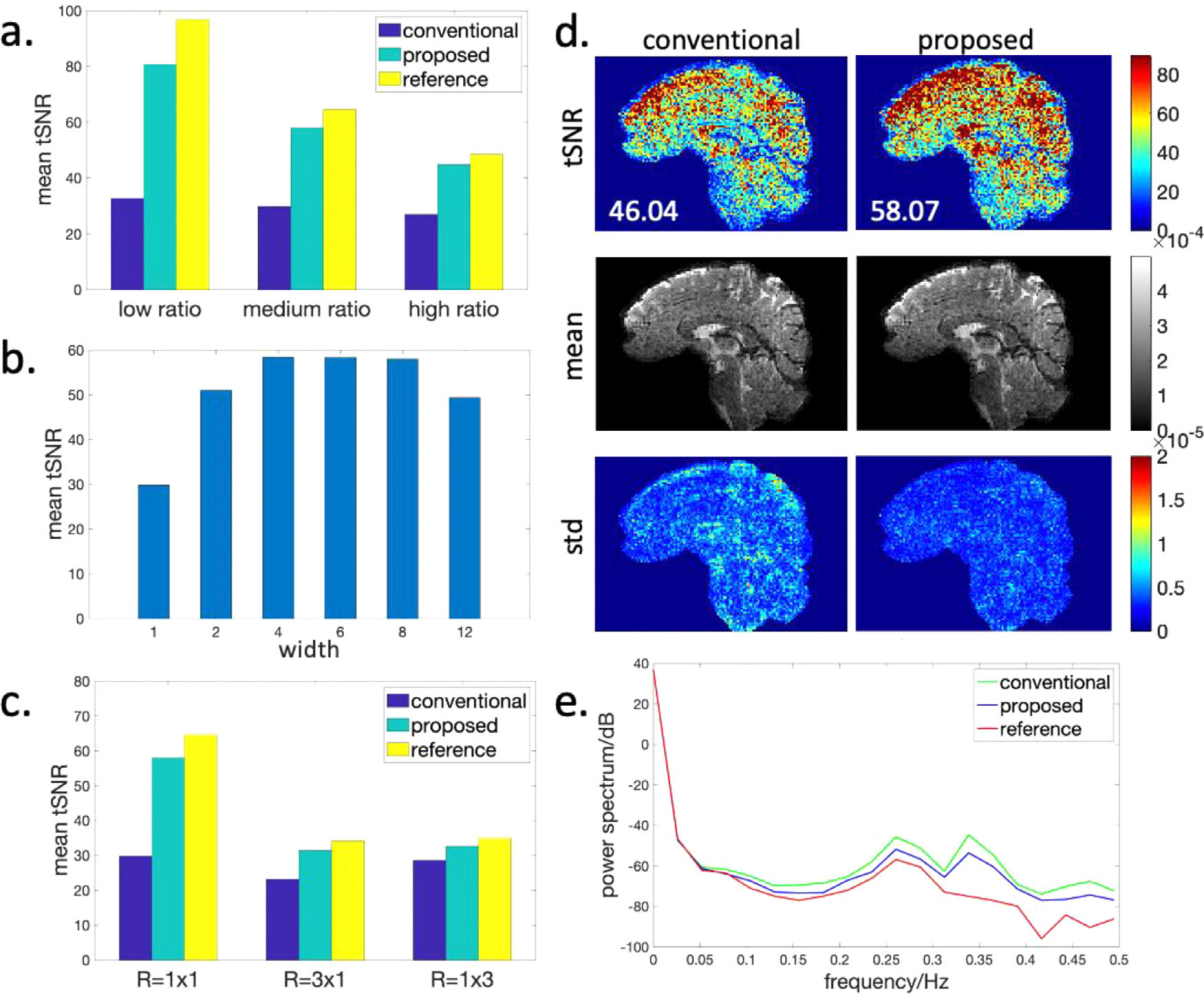
The reconstruction results of the simulation data. The dataset without inter-shot phase variations is used as the reference. (a) Mean tSNR of different methods at varying thermal to physiological noise ratios in the fully sampled regime. Low ratio corresponds to low thermal noise. The proposed method used seg-CAIPI(6,1) sampling trajectory. (b) Mean tSNR of the proposed method with different choices of the width parameter at the medium thermal noise level. The conventional method corresponds to width=1. The $\Delta k_{z}$ blip was kept to be 1. (c) Mean tSNR of different methods at varying acceleration factors at the medium thermal noise level. The proposed method used seg-CAIPI(6,2) at R=1×3 and 3×1. (d) Maps of tSNR (mean value shown on the bottom left), temporal mean magnitude and temporal standard deviation of the conventional method (left) and the proposed method (right) in a thermal noise-free regime. The proposed method used seg-CAIPI(8,2) sampling trajectory. The acceleration factor is R=2×4. (e) The mean power spectrum across the masked brain of the results shown in (d).

Fig. 3 shows the results of the simulation experiments. Fig. 3a compares the mean tSNR of the conventional and proposed methods at low/medium/high thermal to physiological noise ratios in a fully sampled regime. The proposed method achieves a significantly higher tSNR than the conventional method in all 3 cases, which approaches the thermal noise only reference. As expected, the relative tSNR differences between different methods and the reference data reduce when thermal noise increases. Fig. 3b and c show further investigation at medium thermal noise level. Fig. 3b shows the impact of the parameter width on the performance of the proposed method. Fig. 3c shows the impact of acceleration factor. Fig. 3d show the tSNR, temporal mean and temporal standard deviation maps of the simulation results at R=2×4 in a thermal noise-free regime. Fig. 3e shows the mean power spectrum of the reconstructed time series across the masked brain of the results in Fig. 3d. Compared to the reference data which is free from inter-shot phase variations, the conventional 3D multi-shot EPI method has higher noise level across a broad frequency range, and the proposed method reduces the apparent broadband physiologically induced noise. The sampling masks of all under-sampled datasets are shown in supplementary Fig. S3. More simulation experiments validating the ability of the proposed method to recovery phase variations can be found in supplementary Fig. S4.

**Fig. 4:**
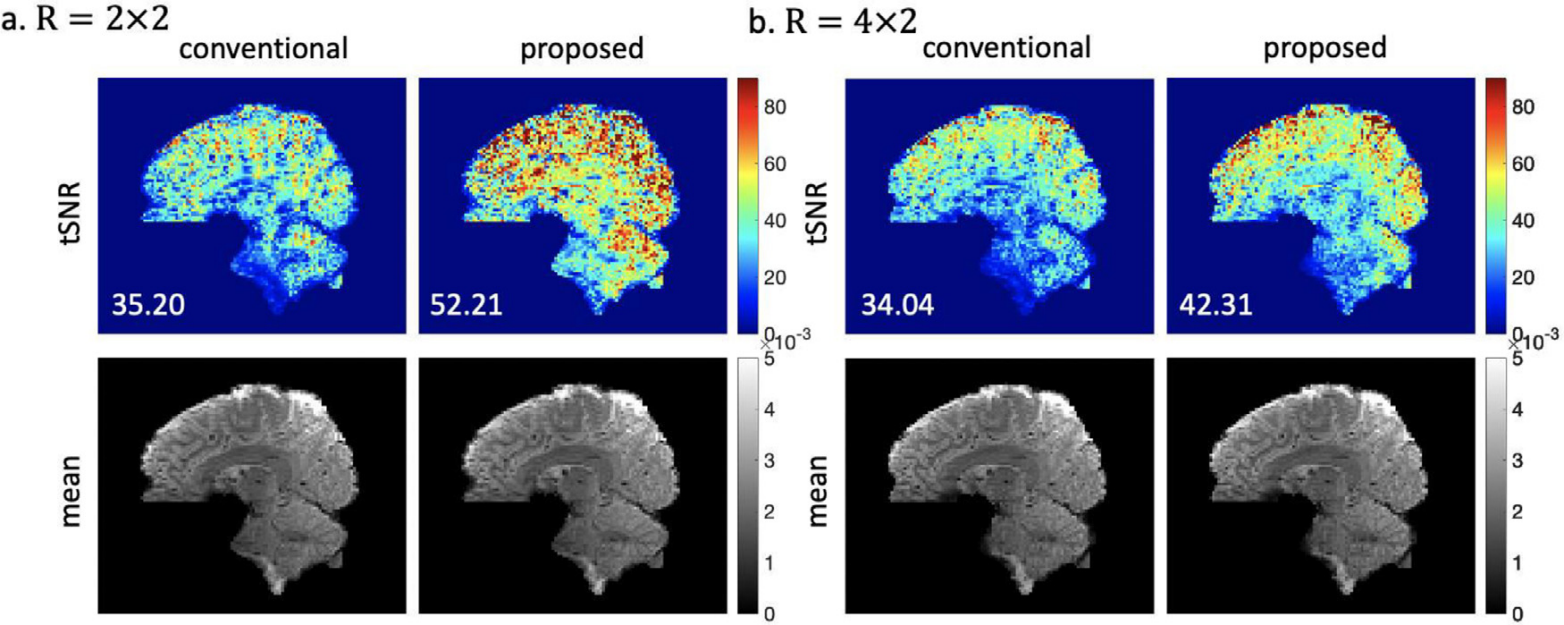
The reconstruction results of 1.8mm isotropic resolution in-vivo datasets acquired at (a) R=2×2 and (b) R=4×2 respectively. The proposed method used seg-CAIPI(8,3) sampling trajectory. Mean tSNR across the whole 3D brain is shown on the bottom left for each tSNR map.

Fig. 4 shows the tSNR results of the 1.8mm isotropic resolution 3D EPI datasets with under-sampling factors $R_{y}=2/4$ and $R_{z}=2$ at resting-state. In both cases, the proposed method achieves significantly higher tSNR than the conventional 3D method. Note the tSNR of conventional method does not improve significantly as $R_{y}$ decreases, which indicates that the higher SNR at R=2×2 was negated by the higher physiological noise compared to R=4×2. Accordingly, the proposed 3D method achieves a larger tSNR gain at R=2×2, resulting in considerably higher tSNR at lower $R_{y}$. It is also worth noting the proposed 3D method achieves even higher tSNR at R=4×2 than the conventional method at R=2×2.

Table 2 shows the comparison of mean tSNR between the conventional and the proposed 3D methods as well as the 2D SMS-EPI method on the resting-state datasets of 5 subjects at 1.8mm isotropic resolution with R=2×4. In this case, the difference in tSNR between the conventional 3D method and SMS-EPI is small and the proposed 3D method only achieves a small improvement over the conventional approach. This tSNR improvement is smaller compared to the tSNR improvement at R=2×2, which indicates that fewer shots per volume can result in lower physiological noise and higher thermal noise. As shown in Fig. 4 and Table 2, the tSNR gain of the proposed method varies depending on the acceleration factor $R_{y}$ and $R_{z}$, despite the same spatial resolution. This is likely because the acceleration factor alters physiological noise by influencing the sensitivity to physiological variations as well as thermal noise by changing the g-factor.

**Table 2:** The mean tSNR of different methods on the resting-state datasets at 1.8mm isotropic resolution of 5 subjects. The acceleration factor isR=2×4. The proposed method used seg-CAIPI(8,2) sampling trajectory.

subject #	conventional 3D	proposed 3D	SMS-EPI
1	32.29	35.48	38.36
2	34.51	37.37	38.62
3	30.16	34.93	33.11
4	31.19	28.56	29.71
5	36.19	35.64	35.02
average	32.87	34.40	34.96

**Fig. 5:**
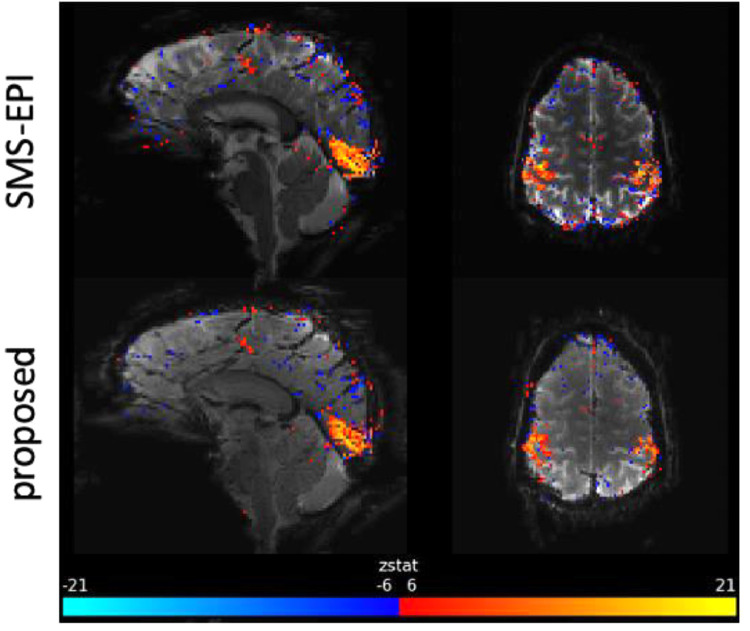
Representative activation maps of the task fMRI experiment at 1.8 mm isotropic resolution by both the proposed 3D and SMS-EPI methods of subject 3 in Table 3.

Table 3 compares the max and mean z-statistics between the proposed 3D and SMS-EPI methods in the task fMRI experiment of 6 subjects at 1.8 mm isotropic resolution with R=2×4. The ROI was independently defined for each subject over motor and visual cortices. Fig. 5 shows the activation z-statistic maps of subject 3. The proposed 3D and SMS-EPI methods show comparable max and mean activations in this case.

**Table 3:** The max z-statistic and mean z-statistic within the selected ROI for the proposed 3D method and the SMS-EPI method in the task fMRI experiment at 1.8mm isotropic resolution of 6 subjects. The acceleration factor is R=2×4. The proposed method used seg-CAIPI(8,2) sampling trajectory.

subject #	max z stats		mean z stats	
	proposed 3D	SMS-EPI	proposed 3D	SMS-EPI
1	**23.09**	21.70	**6.56**	6.01
2	**18.52**	14.86	3.84	**4.02**
3	20.19	**20.70**	5.66	**6.68**
4	17.39	**19.32**	3.43	**5.77**
5	17.46	**21.63**	4.59	**7.49**
6	**22.33**	22.09	7.71	**8.27**

**Fig. 6:**
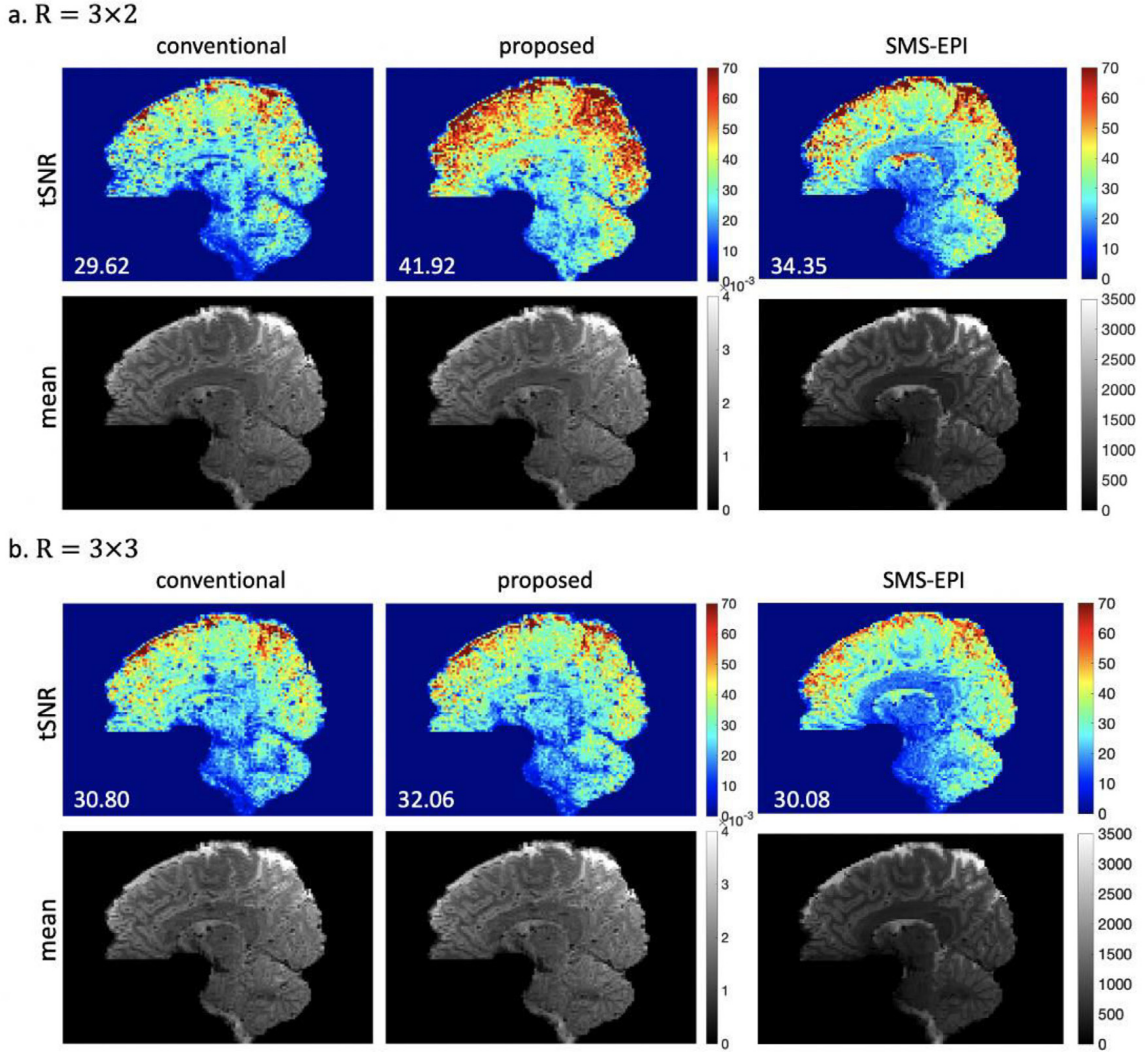
The reconstruction results of 1.5mm isotropic resolution in-vivo datasets acquired at (a) R=3×2 and (b) R=3×3. The proposed method used seg-CAIPI(8,3) at R=3×2 and seg-CAIPI(6,2) at R=3×3. Mean tSNR across the whole 3D brain is shown on the bottom left for each tSNR map.

Fig. 6 shows the results of the 1.5mm isotropic resolution datasets with different acceleration factors $R_{z}=2/3$ and $R_{y}=3$. When $R_{z}=2$, the proposed 3D method achieves a significant improvement in mean tSNR compared to the conventional 3D method, which enables the 3D imaging method to have higher tSNR than SMS-EPI. When $R_{z}=3$, the proposed method achieves a moderate improvement compared to the conventional 3D method, which is comparable to SMS-EPI in this regime. Similar to the results shown in Fig. 4, the tSNR of conventional method does not improve as $R_{z}$ decreases, which is likely again due to the limiting impact of physiological noise. In contrast, the proposed 3D method achieves a significantly higher tSNR when $R_{z}$ is reduced, demonstrating that the impact of physiological noise has been greatly reduced at R=3×2.

**Fig. 7:**
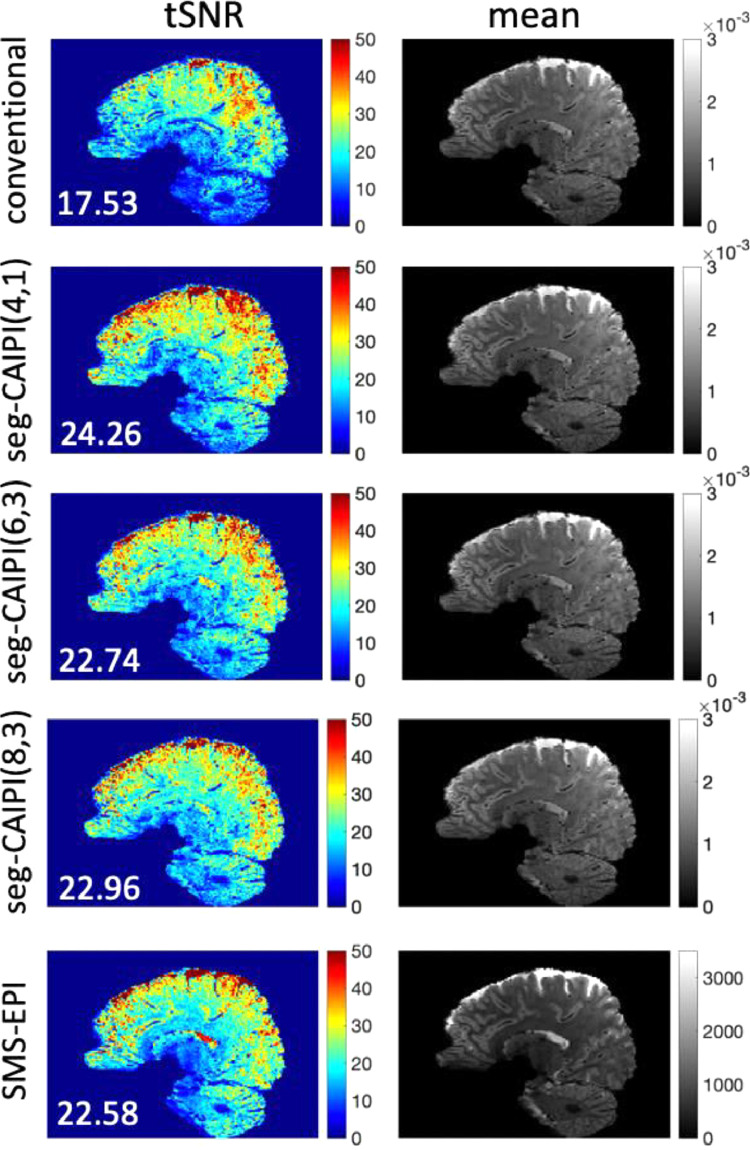
The reconstruction results of 1.2mm isotropic resolution in-vivo datasets. The seg-CAIPI acquisitions with different choices of width were compared. The acceleration factor is R=3×2. Mean tSNR across the whole 3D brain is shown on the bottom left for each tSNR map.

Fig. 7 shows the reconstruction results of the 1.2mm isotropic resolution datasets with varying 3D acquisitions, where we compare the proposed method with width=4/6/8 (corresponding to 2/3/4 shot groups), along with the conventional 3D method and SMS-EPI. All three seg-CAIPI datasets achieve a mean tSNR comparable to SMS-EPI, which is much higher than the conventional approach. The highest tSNR was achieved with width=4 in this case, while the choice of width leads to a moderate difference. We also show a comparison in the impact of width parameter between different resolutions in supplementary Figs. S5 and S6.

**Fig. 8:**
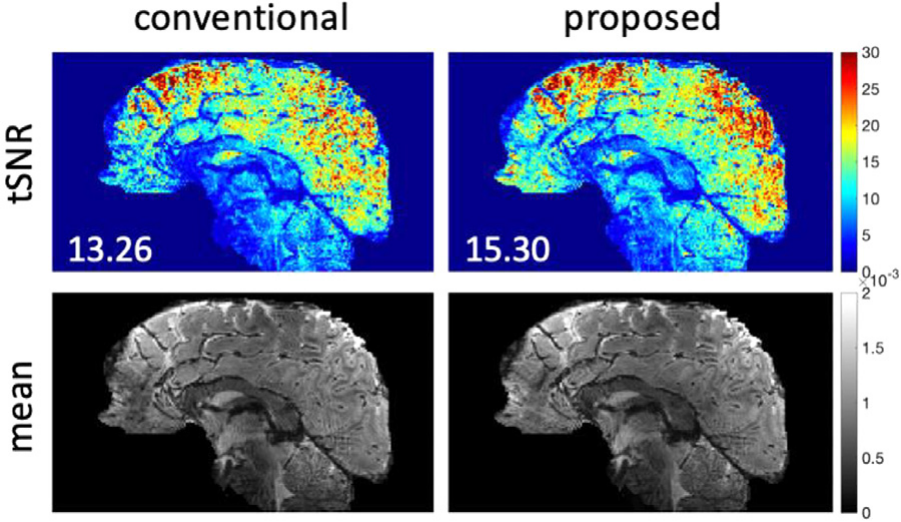
The reconstruction results of 1mm isotropic resolution in-vivo dataset. The proposed method used seg-CAIPI(6,3) sampling trajectory. The acceleration factor is R=4×2. Mean tSNR across the whole 3D brain is shown on the bottom left for each tSNR map.

Fig. 8 shows the reconstruction results of the 1mm isotropic resolution datasets. The proposed method achieves a moderate improvement in mean tSNR compared to the conventional method. At this high resolution, tSNR values are relatively low, and the improvement over the conventional 3D method is limited by the thermal noise dominance. However, there does appear to be a localized improvement in tSNR in the cerebellum, for example, consistent with greater impact of respiratory phase fluctuations nearer to the lung cavity. Another experiment with comparison to SMS-EPI at 1.05mm isotropic resolution is shown in supplementary Fig. S7. Note part of the differences in tSNR between 3D EPI and 2D SMS-EPI in this case can be accounted for by the mismatch in the in-plane acceleration factor. With that being considered, we expect that the 3D EPI and 2D SMS-EPI have overall comparable tSNR at around 1mm isotropic resolution, which is consistent with previous report (Huber et al., 2018).

## 4 Discussion

In this paper, we present a method to improve the robustness of 3D multi-shot EPI acquisition to physiological fluctuations for fMRI at 7T, which incorporates the use of a 3D seg-CAIPI sampling scheme and a reconstruction method based on Hankel structured low-rank matrix completion. In-vivo experiments have demonstrated that the proposed 3D method outperforms the conventional 3D method across a wide range of isotropic resolutions from 1mm to 1.8mm and varying acceleration factors. The tSNR improvement gained by the proposed 3D method is particularly significant where the conventional 3D method suffers from a great tSNR loss compared to 2D SMS-EPI imaging, suggesting the high impact of physiological noise. With the proposed method, 3D EPI imaging can achieve a tSNR comparable or higher than 2D SMS-EPI. Thus, the proposed method could increase the utility of 3D EPI in regimes currently dominated by 2D multi-slice methods.

The performance of the proposed method in terms of its ability to boost tSNR is related to physiological to thermal noise ratio, which is difficult to characterise and affected by many factors jointly, among which we focus on spatial resolution and acceleration factor. In general, the proposed method is more beneficial in higher SNR regimes e.g., either low spatial resolution or low acceleration factor given that other conditions are the same, where the SNR benefit obtained by higher signal strength or more data available could be negated by higher physiological noise. Although the tSNR gain varies in different sampling protocols, the proposed method is beneficial in most cases where the inter-shot phase variations play a non-trivial role in limiting the tSNR. The proposed method could also be less effective as the conditioning gets worse at very high acceleration factor. Supplementary Fig. S8 shows an example where the proposed method fails to improve tSNR at *R* = 4 × 4 and 1.8mm isotropic resolution.

The seg-CAIPI trajectory is a combination of interleaved sampling along the shot dimension and CAIPI blipping. Similar to other 3D EPI blipped-CAIPI schemes (Narsude et al., 2016; Stirnberg and Stocker, 2021; Wang et al., 2019), seg-CAIPI acquires 3D k-space using a series of blipped, band-limited (in $k_{z}$) readouts, except that it returns to acquire different samples in the same “band” in different interleaves. Also, each shot of the seg-CAIPI sampling covers a wider band along $k_{z}$ compared to its conventional alternatives, as this sampling pattern aims to optimize the sampling trajectory of each shot group in addition to the overall shot-combined sampling pattern. This seg-CAIPI sampling pattern is used to facilitate the joint reconstruction of images for each shot group, instead of a single shot-combined image that suffers from phase cancellation, which is the key to the proposed reconstruction. While we observed significantly better performance of the proposed joint reconstruction on the seg-CAIPI data compared to the conventional blipped-CAIPI data with sequential or interleaved ordering (see supplementary Figs. S1&2), the optimal sampling for structured low-rank reconstruction is still a topic of study (Haldar, 2014). Therefore, alternative 3D CAIPI sampling with different shot widths and shot interleaving schemes could be explored as well. It is worth mentioning that although the seg-CAIPI sampling pattern benefits the proposed reconstruction, it may not be optimal for conventional shot-combined SENSE reconstruction. The seg-CAIPI sampling typically achieved lower tSNR than conventional sampling with the same conventional reconstruction in most cases, which is probably due to the interleaved sampling scheme leading to greater signal variations between adjacent k-space samples.

It is important to note that the proposed reconstruction operates on each image volume independently, and only jointly reconstructs shot groups corresponding to a single volume. Hence, it reduces the vulnerability to physiological variations within the acquisition window of each volume, while retaining full temporal degrees of freedom across different volumes and leaving the BOLD-related signal fluctuations and temporal characteristics intact. Similarly, the proposed method cannot remove other inter-volume fluctuations, except for what results from the intra-volume variations. Furthermore, it deals with the inter-shot phase variations without requiring any information or external measurements of the physiological traces, and only requires the condition that the phase fluctuations are temporally coherent with respect to the shot-to-shot TR. Also, although images of all the shot groups are sum-of-squares combined in the proposed method, other image combination approaches which preserve the phase information in the final output image can also be considered for applications where complex data are necessary.

Here, we reduce the temporal scale of the physiological variations from the acquisition window of each volume to each shot group by reconstructing an individual image for each shot group. Thus, there is a trade-off between the number of shot groups and the amount of data available for each group. Increasing the number of shot groups can enhance the intra-shot group consistency, but may make the reconstruction more challenging due to the reduced number of samples for each shot group. In comparison, decreasing the number of shot groups might suffer from strong phase variations within the shot group that impairs the efficacy of the proposed reconstruction. Note the minimal TE achievable is limited by the largest $k_{z}$ rewind blip of the seg-CAIPI acquisition, which could be another consideration limiting high width parameters, and thus the number of shot groups, while width≤8 used in this work does not prolong the minimal TE compared to the standard blipped-CAIPI sampling. In this work, 2-4 shot groups have been demonstrated to be effective across the 3D whole brain imaging protocols tested, which are accomplished by width=4,6or8. In general, using more shot groups is more beneficial in low spatial resolution, low acceleration regimes (see supplementary Figs. S5 and S6). At 1.8mm isotropic resolution, we recommend 4 shot groups (width=8). At 1.2mm isotropic resolution, this choice does not make a significant difference. Extending the current framework to hybrid radial-Cartesian sampling like 3D TURBINE (Chiew et al., 2016) may allow for more flexibility to choose the optimal binning window for each shot group retrospectively, as the golden angle scheme can provide near-uniform coverage at any window size. Radial sampling also has intrinsic robustness to temporal fluctuations, which may also benefit the robustness of 3D sampling further.

The proposed approach is based on MUSSELS, a method using Hankel structured low-rank matrix completion to reconstruct multi-shot diffusion weighted images. The idea of structured low-rank matrix completion has also been successfully employed in some other applications such as calibration-less parallel imaging reconstruction (Haldar and Zhuo, 2016, Liu et al., 2021, Shin et al., 2014, Yi et al., 2021), EPI Nyquist ghost correction (Lee, Jin and Ye, 2016a, Lobos et al., 2018, Lobos et al., 2021), and trajectory error correction (Mani et al., 2018). The inherent linear dependency of the phase-corrupted multi-shot data has enabled us to leverage the low-rank constraint on its block-Hankel matrix representation, assuming image phase fluctuations driven by respiration are relatively smooth in the spatial domain, an assumption which has been employed in other work (Wallace et al., 2020). A future extension of this work could be the joint reconstruction across multiple volumes to improve the conditioning of the reconstruction in highly under-sampled regimes, but special care should be taken not to alter the temporal degrees of freedom of the time series. Also, initializing the current intra-volume reconstruction with data across multiple volumes could be a promising option for improving convergence.

The reconstruction strategy employed here empirically estimates the hyperparameter λ which trades off between the data consistency term and the low-rank constraint term. The parameter was hand tuned to optimize for the output tSNR, which provides a useful heuristic for hyperparameter tuning in this problem without requiring any additional training data or prior knowledge. Note the proposed reconstruction works on each volume independently, while the tSNR is a measurement over the entire time course which reflects both the temporal variations and the fidelity of the image magnitude strength. In this work, under-regularization results in high temporal standard deviation and over-regularization could lead to either high temporal standard deviation or a reduced image magnitude which prevents the tSNR from increasing despite the low temporal standard deviation, depending on the acceleration factor (see supplementary Fig. S9). The acquired data is normalized before reconstruction and the optimal λ is very robust (3E-4 or 6E-4) across all the in-vivo datasets.

The proposed reconstruction incorporates the low-rank constraint in the SENSE-based parallel imaging formulation which uses coil sensitivities explicitly, derived from a separate multi-shot calibration dataset in this work. Compared to calibration-free formulations which construct the block-Hankel structured matrix from the multi-coil k-space, the calibration-based formulation which reconstructs a single coil k-space is more computationally efficient as the block-Hankel structured matrix could have a much smaller size. Also, the coil sensitivity maps are easy to obtain by reference scan, and the moderate phase variations in fMRI typically do not lead to visible artifacts in the calculated coil sensitivities. However, in the worst case scenario where high fidelity coil sensitivity maps cannot be obtained from the calibration data, alternative approaches can be used, such as employing the low-rank tensor representation (Hess et al., 2021; Liu et al., 2021; Yi et al., 2021) of multi-coil k-space for a calibration-less reconstruction without explicit use of coil sensitivity maps, or using a calibration consistency constraint that jointly identifies a coil null space from the imaging and calibration data, without trusting either dataset completely (Lobos et al., 2021). In addition, partial Fourier sampling which is typically used to reduce TE was not employed in this work, but it is also compatible with the seg-CAIPI trajectory and the proposed reconstruction. When partial Fourier sampling is used, virtual conjugate shots (Bilgic et al., 2019) can also be incorporated into the block-Hankel structured matrix construction. This approach was evaluated on the retrospectively under-sampled partial Fourier data (see supplementary Fig. S10), however, the reconstruction results were not further improved compared to the results achieved without using virtual conjugate shots. Simulation experiment suggests that this is possibly because the baseline image phase was not sufficiently smooth to effectively leverage the virtual conjugate shot constraint.

We used the convex nuclear norm minimization to enforce the low-rank property of the block-Hankel structured matrix in this work. However, instead of convex soft thresholding, we used a non-convex hard thresholding in the nuclear norm minimization subproblem in ADMM, as it has shown its ability to achieve better image quality than soft thresholding in this data, particularly in low tSNR regimes at 1mm isotropic resolution, at the cost of slightly lower output tSNR. A comparison of soft and hard thresholding reconstructions is shown in the supplementary Fig. S11.

One limitation of the proposed reconstruction is the computation time. The reconstruction takes ∼120 seconds (3.1 GHz Intel Core i7 and 16 GB RAM) for a 2D 116×96 sagittal matrix at R=2×2. Since all sagittal slices can be reconstructed independently, the reconstruction does parallelize well with additional computational resources. Also, the reconstruction time could probably be further reduced by code optimization, using an SVD-free rank minimization algorithm (Lee et al., 2016b), or a matrix lifting-free algorithm (Ongie and Jacob, 2017). Another limitation of this work is that no consideration of motion artifacts is taken into account. As the low-rank property of the block-Hankel matrix representation relies on the assumption that different shot groups share the same image magnitude, motion induced magnitude mismatch between shot groups could violate this assumption. Thus, a future extension of this work could be to incorporate motion estimates in the forward model to improve the low-rankness of the block-Hankel matrix representation and ultimately the robustness of 3D multi-shot EPI reconstruction further. We anticipate that one particularly useful application of the proposed method is in brainstem and spinal cord imaging, where multi-shot 3D imaging can provide the high spatial resolution needed to resolve important structures, but where respiration induced phase variations are expected to be more significant, and motion artifacts could also be more severe.

## 5 Conclusion

In this work, we proposed to use the low-rank constrained reconstruction on the data acquired with the 3D segmented CAIPI sampling pattern, which is demonstrated to be less vulnerable to inter-shot phase variations and thus improves the robustness of 3D multi-shot EPI for fMRI at 7T.

## CRediT authorship contribution statement

**Xi Chen:** Methodology, Software, Investigation, Writing – original draft. **Wenchuan Wu:** Investigation, Writing – review & editing. **Mark Chiew:** Conceptualization, Investigation, Writing – review & editing.

## Declaration of Competing Interest

The authors declare that they have no known competing financial interests or personal relationships that could have appeared to influence the work reported in this paper.

## Data Availability

The reconstruction code is available at https://github.com/XChen-p/Multishot-EPI.

## Appendix Supplementary materials

Image, application 1
