# Supplementary material for: Improving robustness of 3D multi-shot EPI by structured low-rank reconstruction of segmented CAIPI sampling for fMRI at 7T
Source: Neuroimage. 2023 Feb 15;267:119827. doi: 10.1016/j.neuroimage.2022.119827 (PMC10933751; doi:10.1016/j.neuroimage.2022.119827)
Supplement: Supplementary file 1 [file mmc1.docx]

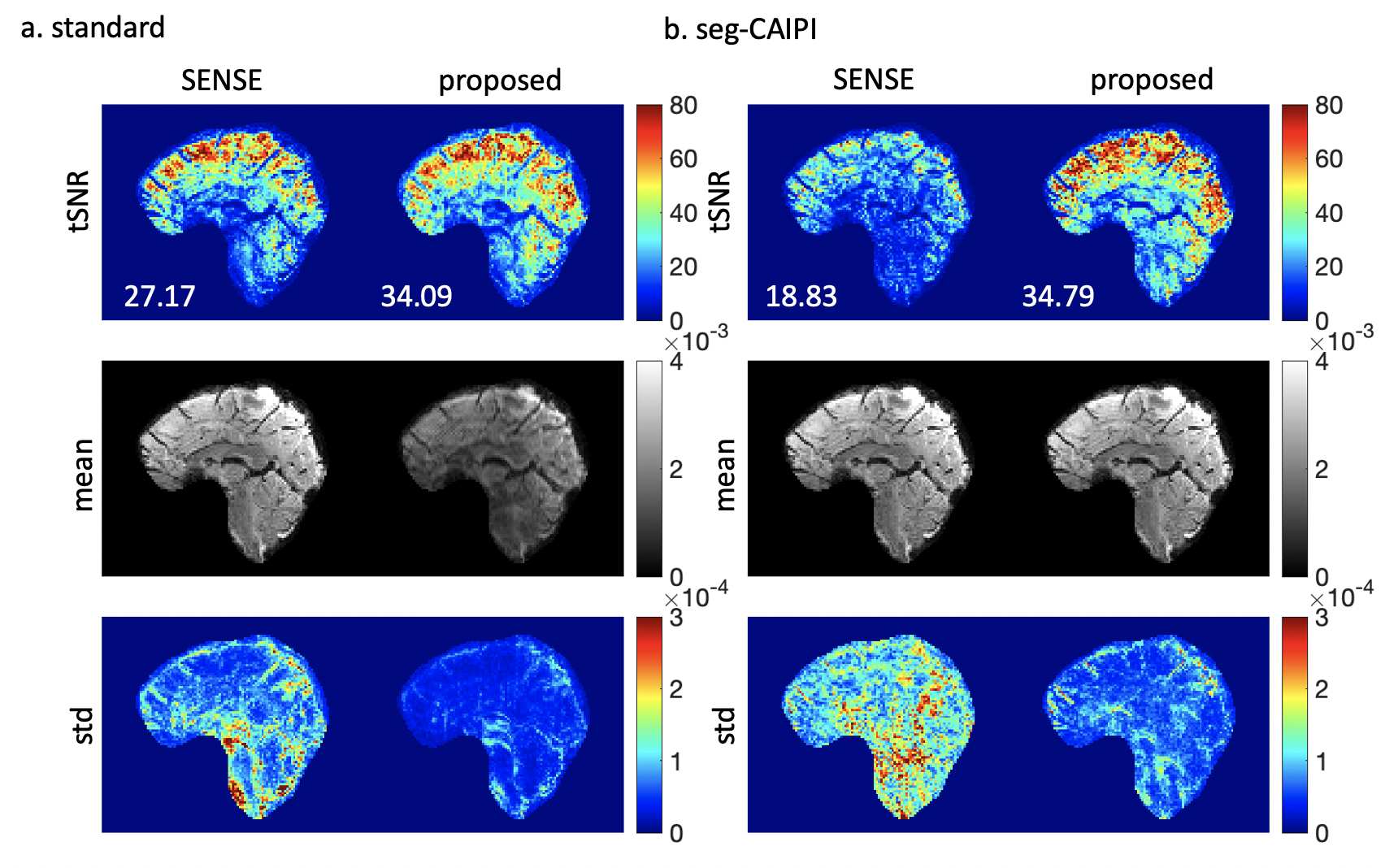


*Figure S1. The comparison of the same reconstructions on in-vivo data acquired with (a) the standard blipped-CAIPI(2,1) and (b) seg-CAIPI(8,3) sampling. These datasets were acquired at* $R=4\times2$ *and 1.8mm isotropic resolution. The proposed reconstruction with 4 shot groups was used for both the standard and seg-CAIPI data. The proposed low-rank reconstruction achieves an tSNR improvement compared to the SENSE reconstruction on the standard sampling data, but its fidelity of the temporal mean image is much lower.* *The mean tSNR calculated for this 2D slice is shown on the bottom left for each tSNR map.*


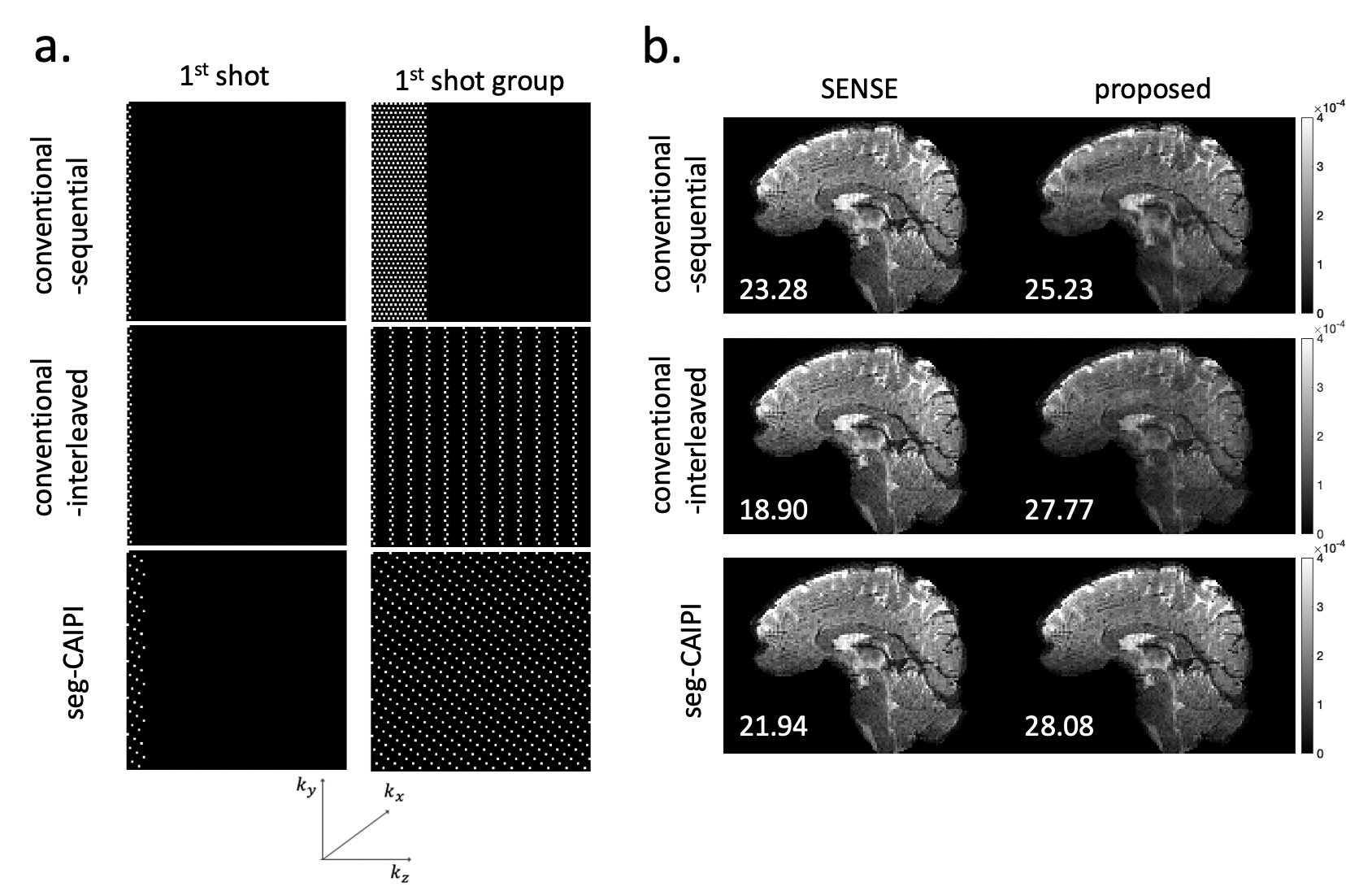


*Figure S2. The comparison of different sampling patterns and their corresponding reconstruction performance on the simulation data. (a) The trajectory of the first shot and first shot group. (b) The temporal mean magnitude image of the conventional SENSE and the proposed reconstructions. The mean tSNR is shown on the bottom left for each temporal magnitude image.* *The standard blipped-CAIPI sampling with sequential ordering is referred to as conventional-sequential and its counterpart with interleaved ordering is referred to as conventional-interleaved. Compared to conventional-interleaved, the seg-CAIPI sampling has a wider span along* $k_{z}$ *for each shot. These simulation datasets were sampled at* $R=2\times2$ *where 4 shot groups with 12 shots per shot group were used.*


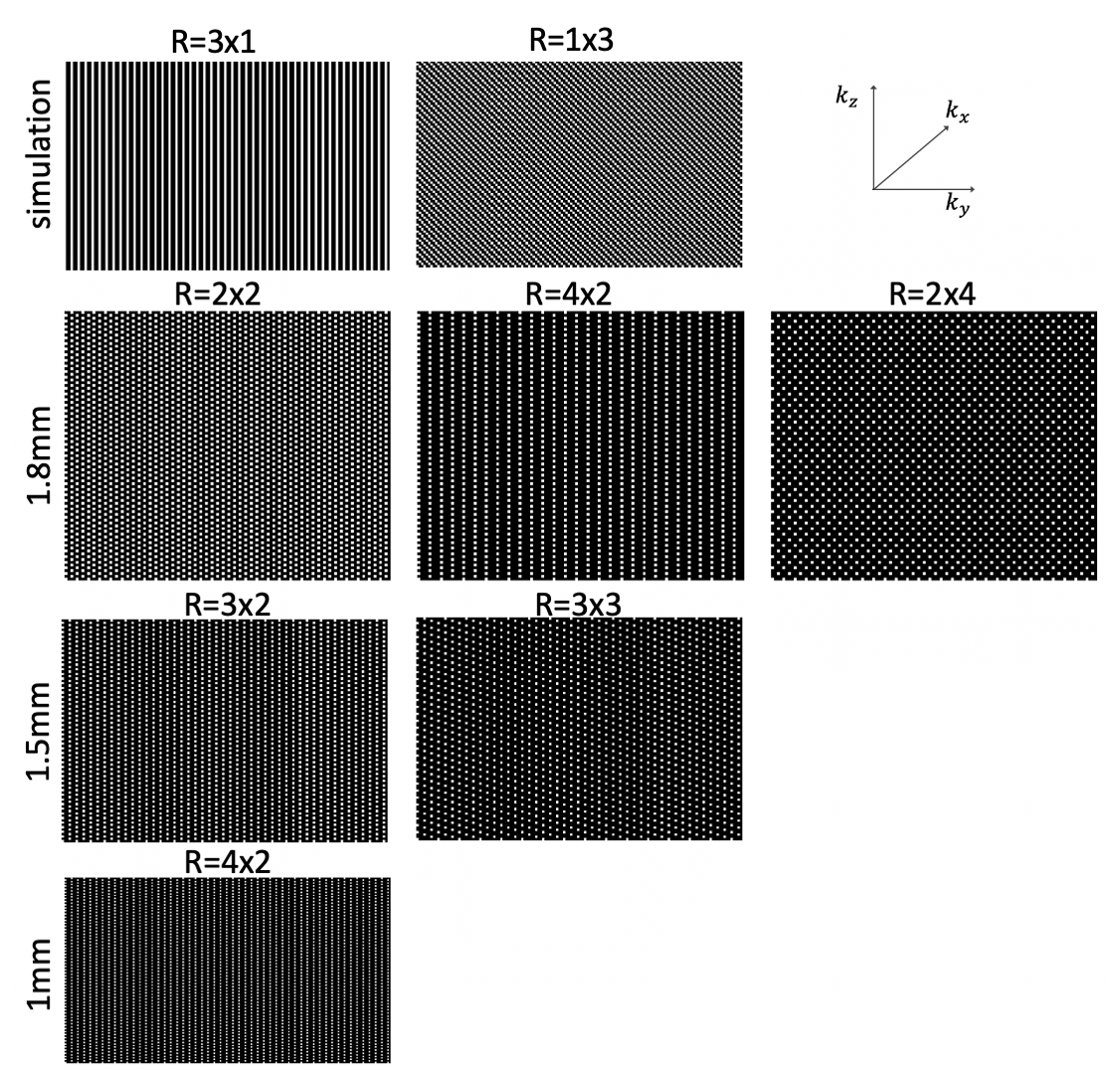


*Figure S3. The overall sampling masks of different datasets. The 1.2mm isotropic resolution datasets used the same sampling mask as the 1.5mm isotropic resolution datasets at* $R=3\times2$ *(different matrix size).*


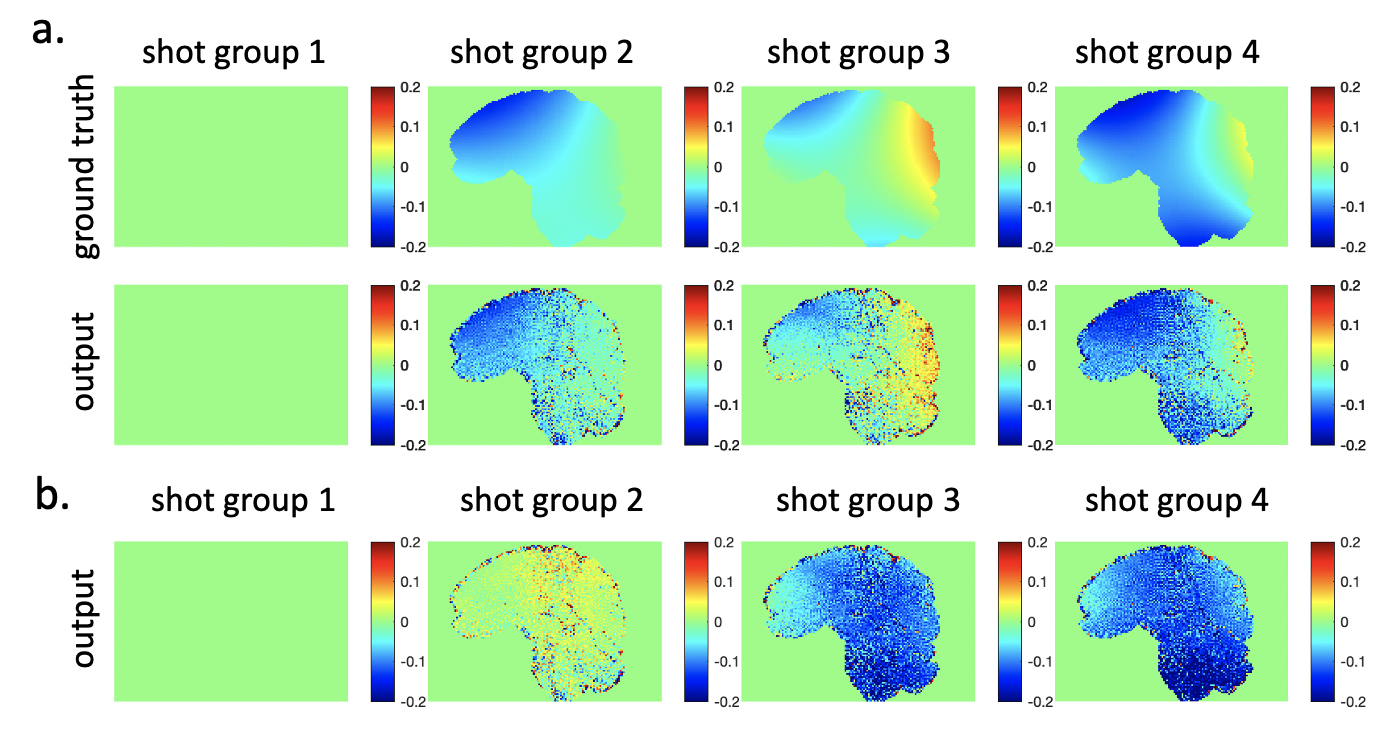


*Figure S4. The phase variation maps of different shot groups reconstructed by the proposed method on the simulation data. The phase difference maps relative to the first shot group are shown for two different datasets: (a) A dataset with inter-shot group phase variations only, where the ground truth phase for each shot group is available. The phase variations of the ground truth (first row) and the reconstruction output (second row) are shown, highlighting good correspondence of the recovered phase variations to the ground truth modulations. (b) A dataset with shot-to-shot phase variations, so the ground truth phase of each shot group is not available. However, distinct "average" phase modulations per shot-group are still recovered. The seg-CAIPI(8,3) sampling pattern at* $R=2\times2$ *was used.*


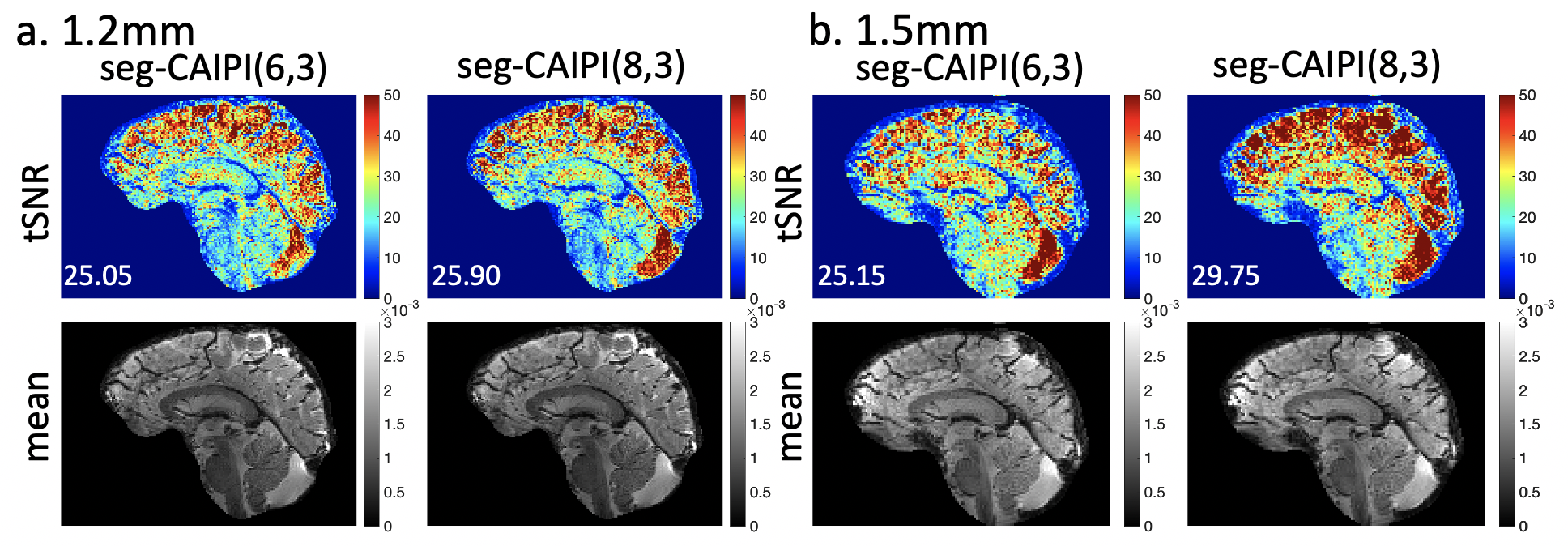


*Figure S5. The impact of parameter width on the performance of the proposed method on the (a)1.2mm isotropic resolution in-vivo dataset and (b) 1.5mm isotropic resolution in-vivo dataset.* $R=3\times2$ *was used. At 1.2mm isotropic resolution,* $width=6$ *(3 shot groups) and* $width=8$ *(4 shot groups) have comparable performance, whereas* $width=8$ *achieves a significantly higher tSNR than* $width=6$ *at 1.5mm isotropic resolution. The mean tSNR calculated for this 2D slice is shown on the bottom left for each tSNR map.*


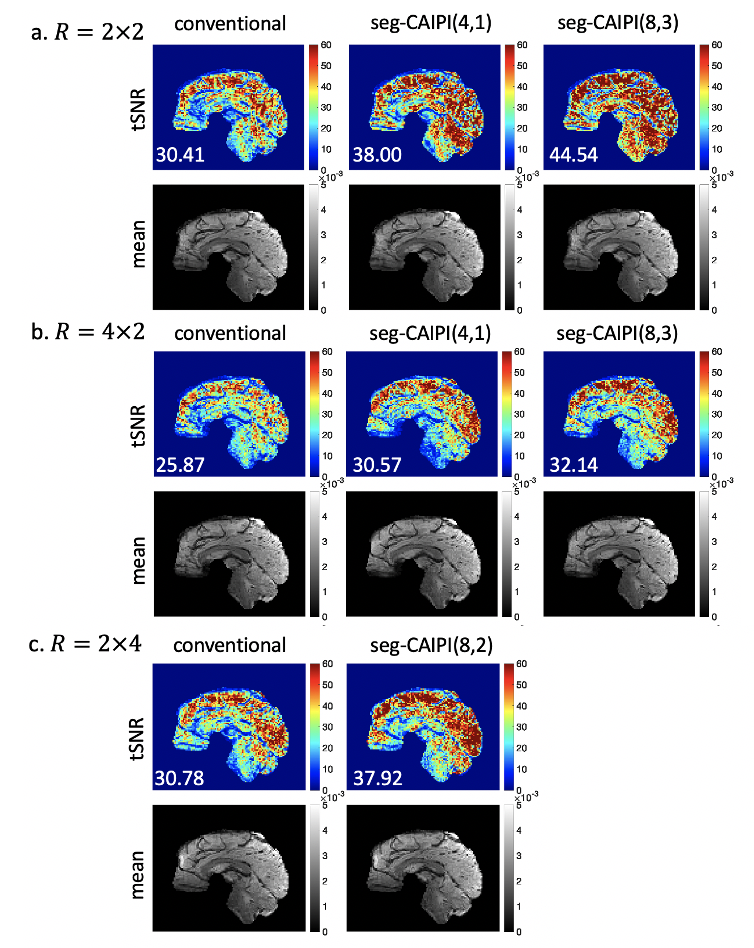


*Figure S6. The reconstruction results of 1.8mm isotropic resolution in-vivo datasets acquired at (a)* $R=2\times2$*, (b)* $R=4\times2$ *and (c)* $R=2\times4$*. The proposed methods with different choices of width were compared at* $R=2\times2$ *and* $R=4\times2$*. At* $R=4\times2$*,* $width=8$ *(4 shot groups) has a slightly higher tSNR than* $width=4$ *(2 shot groups), whereas* $width=8$ *achieves a significantly higher tSNR than* $width=$*4 at* $R=2\times2$*. The mean tSNR calculated for this 2D slice is shown on the bottom left for each tSNR map.*


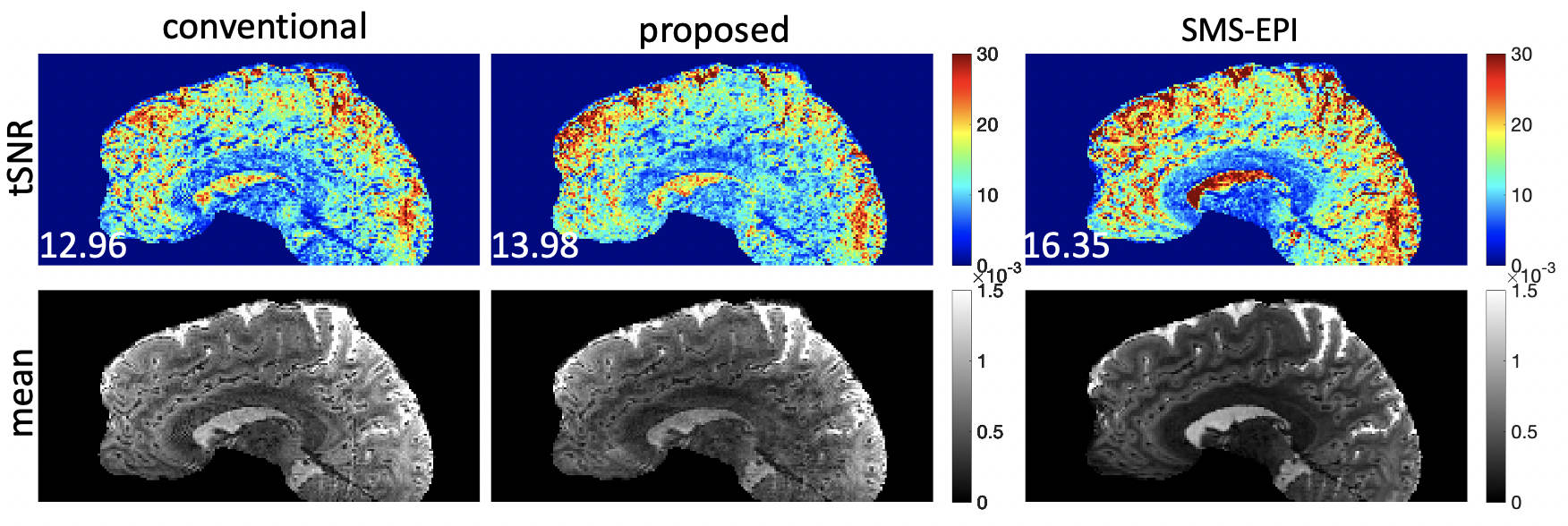


*Figure S7. The reconstruction results of 1.05mm isotropic resolution in-vivo dataset. The conventional and proposed 3D EPI datasets are acquired at* $R_{y}\times R_{z}=4\times2$ *and the SMS-EPI dataset was acquired at* $R_{y}=3$ *and* $MB factor=2.$ *The matrix size was* $200\times200\times96$*. The proposed method used seg-CAIPI(8,3) sampling trajectory. Mean tSNR across the 3D masked brain is shown on the bottom left for each tSNR map. Note the in-plane acceleration factors of 3D-EPI and 2D SMS-EPI are not matched exactly, which partially accounts for the differences in tSNR. It is expected that the tSNR of SMS-EPI with Ry=4 could be lower, thus making 3D EPI and 2D SMS-EPI have overall comparable tSNR at around 1mm isotropic resolution, which is consistent with previous report (Huber et al., NeuroImage 2018).*


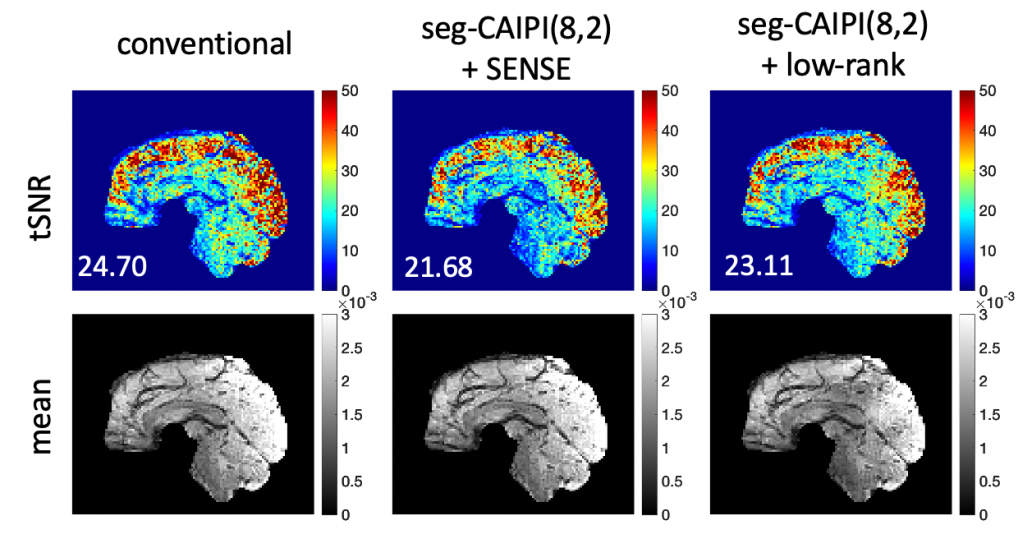


*Figure S8. The reconstruction results of 1.8mm isotropic resolution in-vivo datasets acquired* $R=4\times4$*. The seg-CAIPI data has a lower tSNR than the conventional blipped-CAIPI data with the same SENSE reconstruction, which indicates the impact of physiological noise on the tSNR. The proposed reconstruction on the seg-CAIPI data fails to improve tSNR compared to the conventional method at this acceleration factor. The mean tSNR calculated for this 2D slice is shown on the bottom left for each tSNR map.*


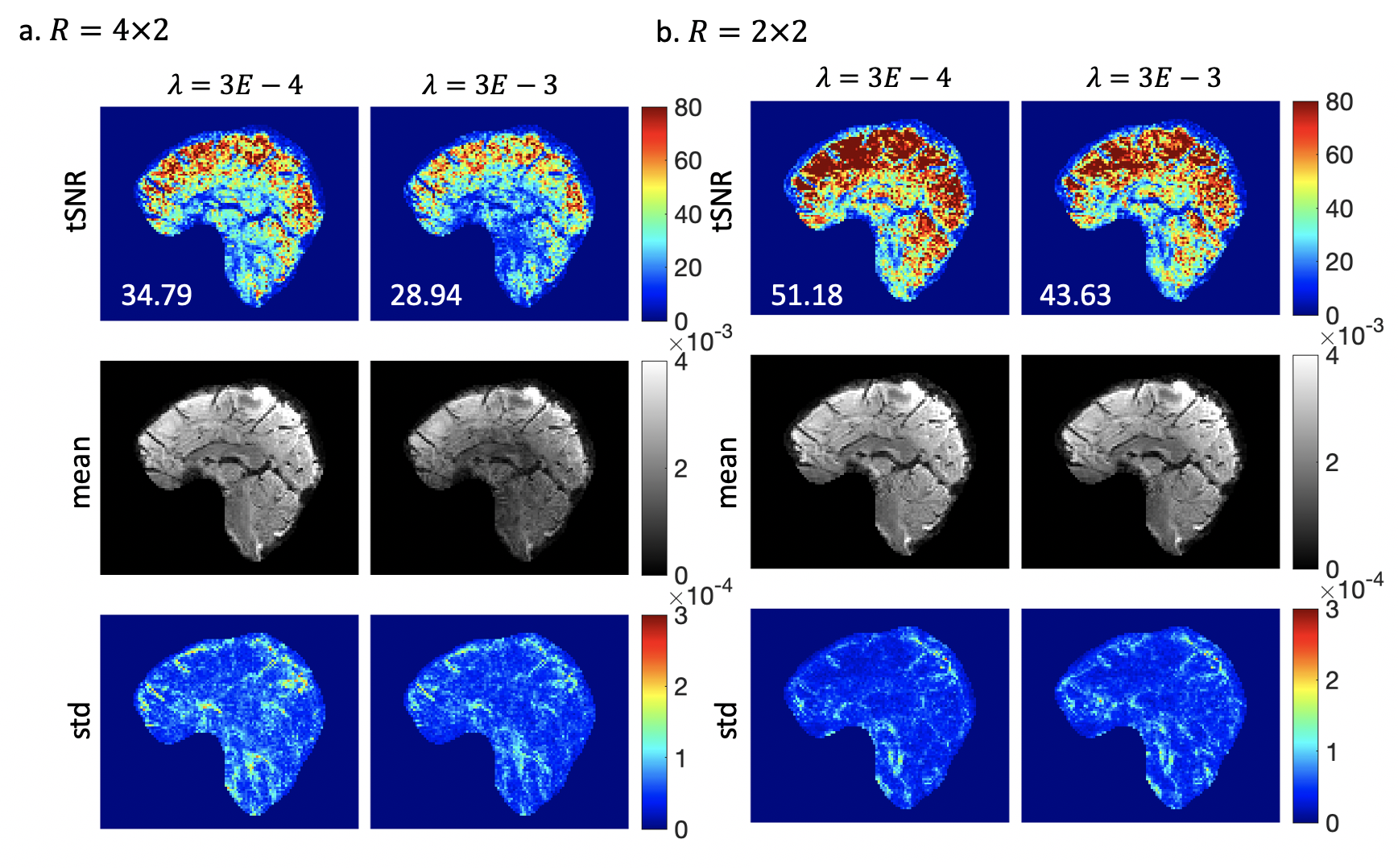


*Figure S9. The impact of over-regularization on the proposed reconstruction on the seg-CAIPI data acquired at (a)* $R=4\times2$ *and (b)* $R=2\times2$*. The optimal* $\lambda$ *is 3E-4, and* $\lambda$*=3E-3 leads to over-regularization. At* $R=4\times2$*, over-regularization can result in biased images which have reduced image magnitude, despite the lower temporal standard deviation than the optimal choice. At* $R=2\times2$*, the fidelity of the temporal mean image achieved by over-regularization is higher, but the temporal standard deviation is higher too. The mean tSNR calculated for this 2D slice is shown on the bottom left for each tSNR map.*


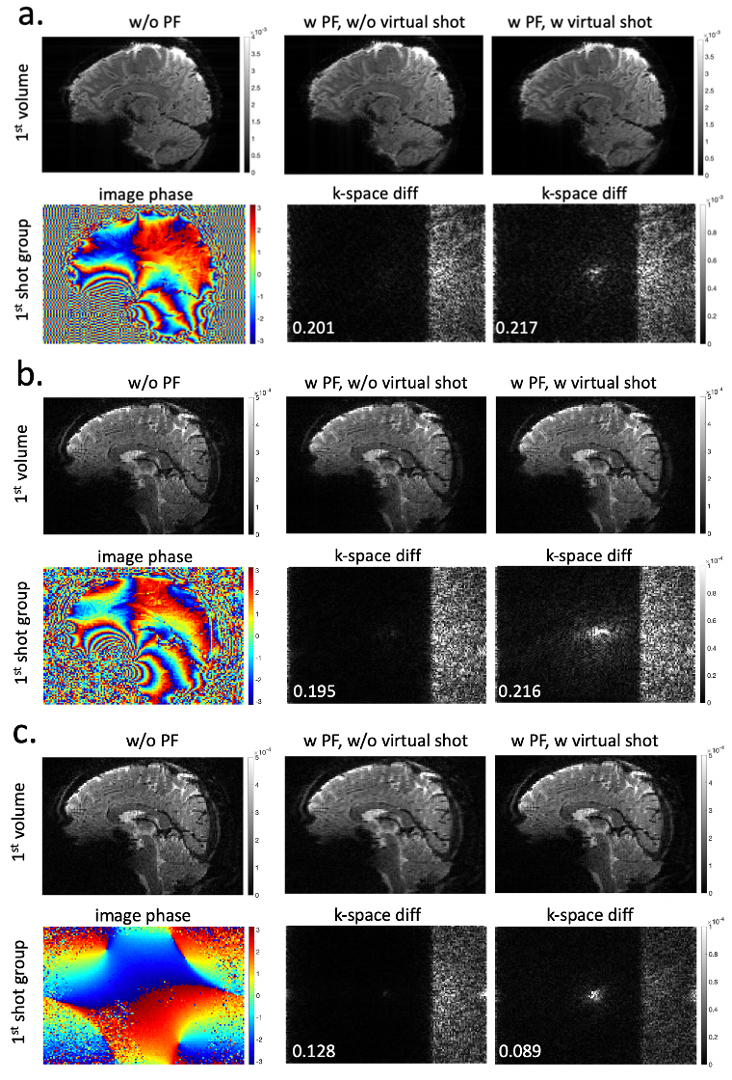


*Figure S10. The comparison of the proposed reconstruction with and without using virtual conjugate shot on (a) a retrospectively partial Fourier sampled in-vivo dataset and (b-c) two partial Fourier sampled simulation datasets with different ground truth image phases. In each subfigure, the reference dataset reconstructed by the proposed method without partial Fourier sampling and virtual conjugate shot is shown in the left column, and the partial Fourier dataset reconstructed without and with virtual conjugate shot approach are shown in the middle and right column respectively. The first row of each subfigure shows the reconstructed image. The second row shows the k-space error map of the first shot group calculated by using the reconstructed reference dataset as ground truth. For the reference dataset itself, the reconstructed image phase is shown instead. The NRMSE of the images reconstructed without and with virtual conjugate shot are shown in the corresponding k-space error map. The proposed reconstruction was improved by the virtual conjugate shot approach only on the simulation dataset in (c) where the baseline image phase is spatially smoother.*

*
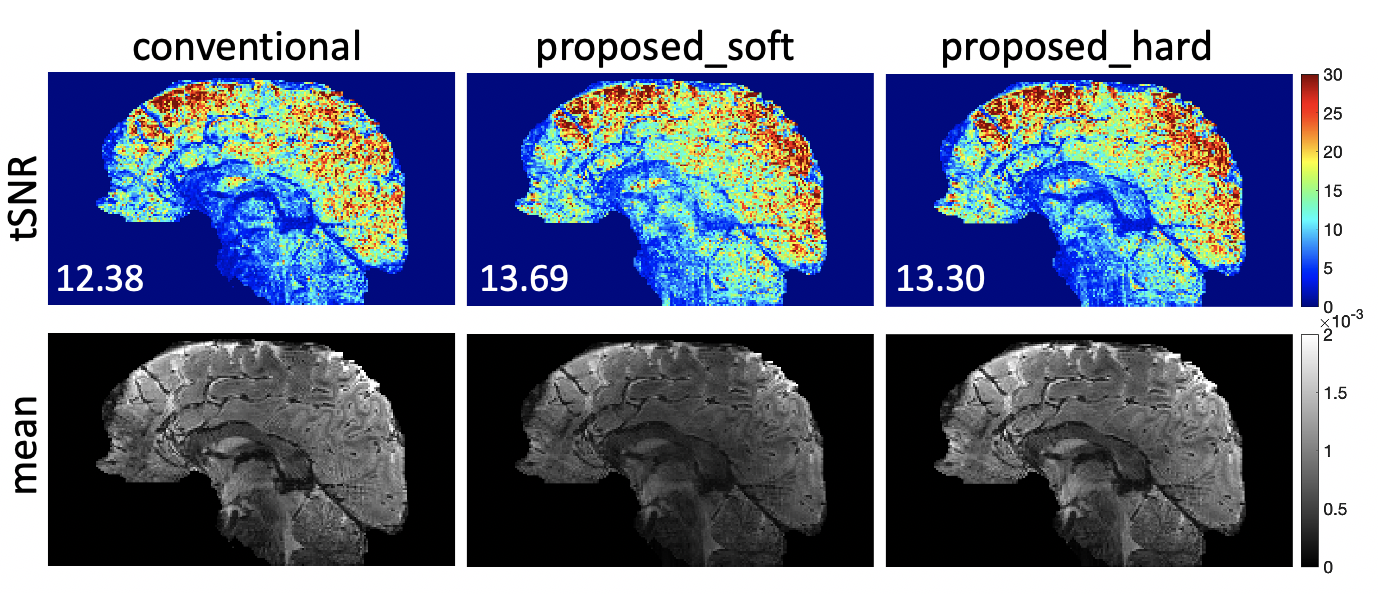
*

*Figure S11. The comparison between soft thresholding and hard thresholding used in the implementation of the proposed reconstruction on the 1mm isotropic resolution dataset at* $R=4\times2$*. Soft thresholding approach leads to reduced image magnitude in the case, whereas hard thresholding approach has higher image fidelity. The mean tSNR calculated for this 2D slice is shown on the bottom left for each tSNR map.*
